# Supplementary material for: Urbanization and Unfavorable Changes in Metabolic Profiles: A Prospective Cohort Study of Indonesian Young Adults
Source: Nutrients. 2022 Aug 14;14(16):3326. doi: 10.3390/nu14163326 (PMC9414085; doi:10.3390/nu14163326)
Supplement: Supplementary file 1 [file nutrients-14-03326-s001.zip › nutrients-1832569-supplementary.pdf]

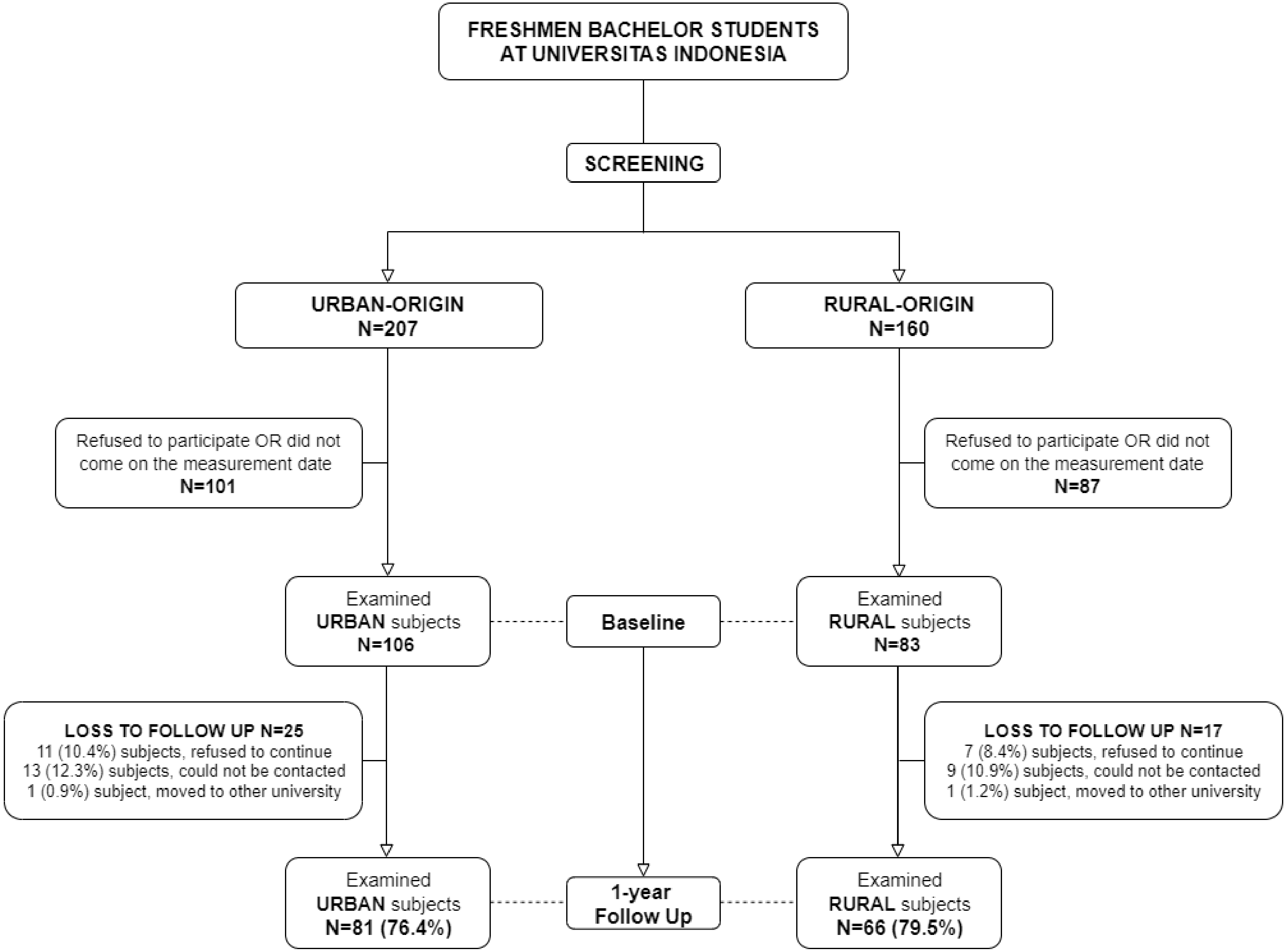

Figure S1. Flow Chart of The Study.

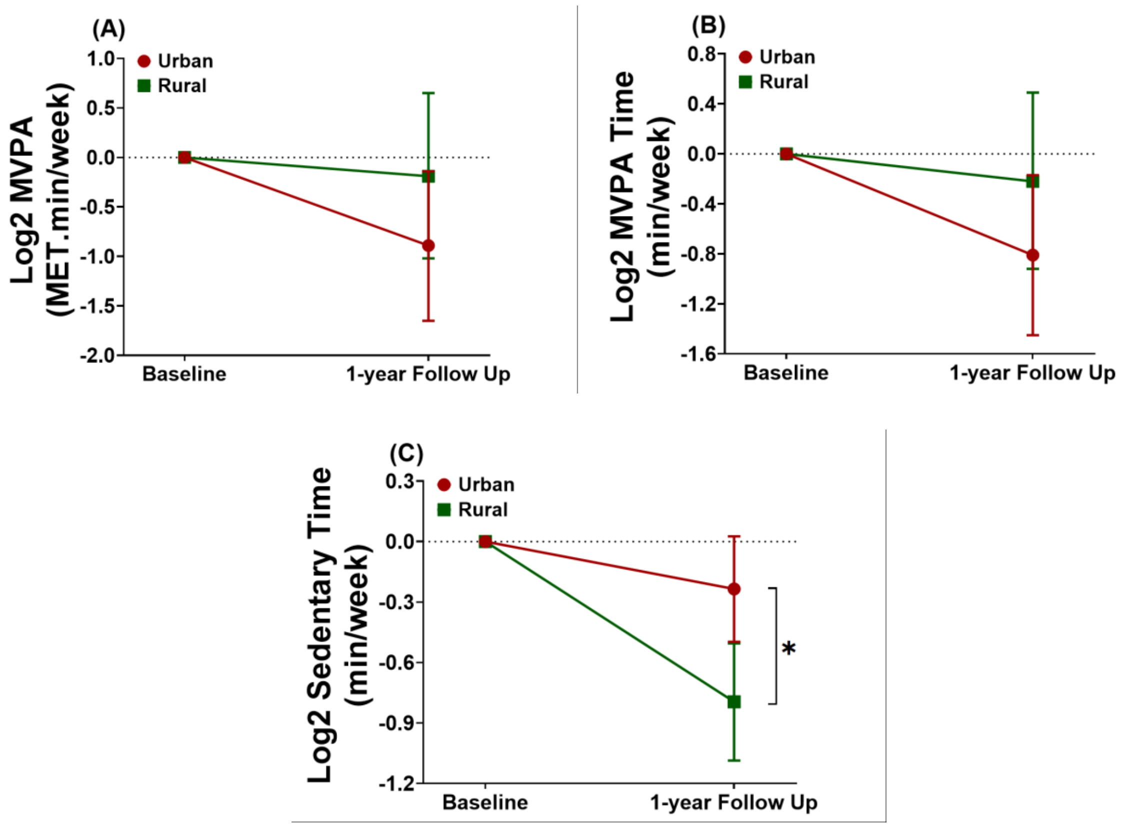

Figure S2. The changes of physical activity levels (A & B) and sedentary time (C) in urban and rural subjects after 1-year of living in an urban environment.

The changes are presented as estimate and its 95% confidence interval and obtained from previously log-transformed variables. The changes in each group and the differences of changes between urban and rural group for each parameter was analyzed using linear-mixed model, adjusted for age and sex. The P-value depicted in the figure represents the P-value for interaction (P_{int}), the level of significance in the differences of changes between the two groups.

*P<0.05

MVPA: moderate-vigorous physical activity

Table S1. Mediation analysis of the effect of dietary intake on the differences of adiposity profiles in urban and rural subjects at baseline.

Model†	Body mass index				Waist circumference				Fat percentage			
	Mean diff. (95%CI)	P value	% changes ††	Indirect effect# (95%CI)	Mean diff. (95%CI)	P value	% changes ††	Indirect effect# (95%CI)	Mean diff. (95%CI)	P value	% changes ††	Indirect effect# (95%CI)
Unadjusted	2.85 (1.60; 4.10)	<0.001			6.42 (3.23; 9.62)	<0.001			5.29 (2.75; 7.83)	<0.001		
Adjusted for age and sex	2.81 (1.55, 4.07)	<0.001			6.37 (3.25; 9.50)	<0.001			5.07 (2.70; 7.44)	<0.001		
(+) Total calories intake	2.66 (1.37; 3.96)	<0.001	-5.3	-0.20 (-0.62; 0.05)	5.99 (2.78; 9.21)	<0.001	-6.0	-0.81 (-2.14; -0.07)	5.05 (2.60; 7.50)	<0.001	-0.4	0.48 (-0.07; 1.19)
(+) Carbohydrate intake	2.88 (1.61; 4.16)	<0.001	+2.5	0.01 (-0.14; 0.17)	6.57 (3.40; 9.75)	<0.001	+3.1	-0.16 (-0.71; 0.18)	5.33 (2.94; 7.73)	<0.001	+5.1	0.48 (-0.18; 1.24)
(+) Fat intake	2.24 (0.97; 3.52)	0.001	-20.3	-0.59 (-1.29; -0.15)	4.95 (1.81; 8.10)	0.002	-22.3	-1.70 (-3.45; -0.47)	4.23 (1.81; 6.65)	0.001	-16.6	-0.52 (-1.41; 0.20)
(+) Protein intake	2.32 (1.00; 3.63)	0.001	-17.4	-0.54 (-1.24; -0.07)	5.22 (1.96; 8.48)	0.002	-18.1	-1.66 (-3.46; -0.43)	4.56 (2.06; 7.06)	<0.001	-10.1	0.11 (-0.81; 0.93)
(+) Fat and protein intake	2.22 (0.92; 3.52)	0.001	-21.0	-0.64 (-1.38; -0.09)	4.95 (1.74; 8.16)	0.003	-22.3	-1.91 (-3.81; -0.55)	4.38 (1.91; 6.85)	0.001	-13.6	-0.09 (-1.21; 0.89)

†All variables as an additional adjustment for age and sex.

††Proportion of changes in mean difference of the model compared to the model adjusted for age and sex only.

#Indirect effect of covariate(s) on anthropometry parameters, obtained by performing bootstrapping with 5000 iterations and presented as its 95% confidence interval.

Table S2. The levels of physical activity and sedentary time measured with GPAQ in urban and rural subjects at baseline.

Variables	Urban<br>N=106	Rural<br>N=83	P values
Total volume of MVPA, MET.min/week	1868	1046	**0.02#**
(geomean, 95%CI)	(1404-2486)	(618-1770)	
Total time spent for MVPA, min/week	423	255	**0.02#**
(geomean, 95%CI)	(328-546)	(164-397)	
Total sedentary time, min/week	441	490	**0.006#**
(geomean, 95%CI)	(411-472)	(444-540)	
Proportion of physical activity intensity categories,			
n (%)			
- Low	18 (17.0)	24 (29.3)	0.12
- Moderate	42 (39.6)	25 (30.5)	
- High	46 (43.4)	33 (40.2)	

# P values derived from linear regression of log transformed data adjusted for age and sex.

The P-values shown in bold represent the statistically significant differences with P<0.05.

GPAQ: Global Physical Activity Questionnaires; MVPA: moderate-vigorous physical activity.

Table S3. Characteristics of study population at 1-year follow-up time.

Variables	Urban N=81	Rural N=66	P values# (adjusted for age and sex)	P values# (adjusted for age, sex, and BMI)
Age, yrs old (mean, SD)	19.4 (0.6)	19.5 (0.7)	0.23	
Sex, n male (%)	31 (38.3)	25 (37.9)	0.96	
BMI, kg/m^2 (mean, SD)	23.7 (5.2)	21.2 (3.3)	**<0.001**	
BMI grouping, n (%)				
- Underweight (<18.5)	8 (9.9)	8 (12.1)	**0.02**	
- Normoweight (18.5-22.9)	34 (42.0)	42 (63.6)		
- Overweight (23-24.9)	13 (16.0)	8 (12.1)		
- Obese (≥25.0)	26 (32.1)	8 (12.1)		
Waist circumference, cm (mean, SD)	81.6 (12.4)	74.5 (8.2)	**<0.001**	
Fat percentage, % (mean, SD)	29.2 (9.0)	25.2 (8.8)	**<0.001**	
FBG, mg/dL (mean, SD)	91.1 (6.7)	90.4 (5.9)	0.56	
HbA1c, % (mean, SD)	NA	NA		
Fasting insulin†, IU/mL	5.2 (4.0-7.0)	2.8 (2.0-3.9)	**0.006**	0.06
HOMA-IR†	1.2 (0.9-1.6)	0.6 (0.5-0.9)	**0.006**	0.07
Leptin†, ng/mL	13.7 (11.3-16.8)	9.7 (7.4-12.7)	**0.01**	0.74
Adiponectin†, µg/mL	4.0 (3.6-4.4)	4.4 (3.9-4.9)	0.28	0.95
Leptin-Adiponectin (L/A) Ratio†	3.5 (2.8-4.3)	2.2 (1.7-3.0)	**0.01**	0.75
Dietary intake, mean (SD)				
- Total calories, kcal	1659 (409)	1510 (422)	**0.04**	0.19
- Fat, gram	69 (25)	60 (21)	**0.03**	0.17
- Protein, gram	58 (16)	55 (17)	0.35	0.85
- Carbohydrate, gram	197 (49)	187 (60)	0.24	0.42

†Not normally distributed continuous variables, presented as geomean (95% CI) and log transformed for analysis.

#Analyzed with linear regression for continuous variables and Chi-square test for categorical variables.

The P-values shown in bold represent the statistically significant differences with P<0.05.

BMI: body mass index; FBG: fasting blood glucose; HOMA-IR: homeostatic model assessment for insulin resistance

Table S4. The effect of the differences in protein intake changes on the differences of BMI increase after 1-year between urban and rural subjects.

Model	ΔBMI††		
	Estimated differences# (95%CI)	P values	Indirect effect (95%CI)
Adjusted for age and sex	0.53 (0.22; 0.84)	<0.001	
Adjusted for age, sex, and Δprotein intake†	0.55 (0.24; 0.86)	<0.001	-0.02 (-0.10; 0.02)

†Δprotein intake = the differences of protein intake at 1-year follow-up time point with protein intake at baseline.

††ΔBMI = the differences of body mass index at 1-year follow-up time point with body mass index at baseline.

#Estimated differences of ΔBMI between urban and rural subjects, analyzed using linear-mixed model.
